# Estimation of type- and subtype-specific influenza vaccine effectiveness in Victoria, Australia using a test negative case control method, 2007-2008

**DOI:** 10.1186/1471-2334-11-170

**Published:** 2011-06-14

**Authors:** James E Fielding, Kristina A Grant, Georgina Papadakis, Heath A Kelly

**Affiliations:** 1 Victorian Infectious Diseases Reference Laboratory 10 Wreckyn Street, North Melbourne, Victoria 3051, Australia; 2 National Centre for Epidemiology and Population Health, The Australian National University Canberra, Australian Capital Territory 0200, Australia

## Abstract

**Background:**

Antigenic variation of influenza virus necessitates annual reformulation of seasonal influenza vaccines, which contain two type A strains (H1N1 and H3N2) and one type B strain. We used a test negative case control design to estimate influenza vaccine effectiveness (VE) against influenza by type and subtype over two consecutive seasons in Victoria, Australia.

**Methods:**

Patients presenting with influenza-like illness to general practitioners (GPs) in a sentinel surveillance network during 2007 and 2008 were tested for influenza. Cases tested positive for influenza by polymerase chain reaction and controls tested negative for influenza. Vaccination status was recorded by sentinel GPs. Vaccine effectiveness was calculated as [(1 - adjusted odds ratio) × 100%].

**Results:**

There were 386 eligible study participants in 2007 of whom 50% were influenza positive and 19% were vaccinated. In 2008 there were 330 eligible study participants of whom 32% were influenza positive and 17% were vaccinated. Adjusted VE against A/H3N2 influenza in 2007 was 68% (95% CI, 32 to 85%) but VE against A/H1N1 (27%; 95% CI, -92 to 72%) and B (84%; 95% CI, -2 to 98%) were not statistically significant. In 2008, the adjusted VE estimate was positive against type B influenza (49%) but negative for A/H1N1 (-88%) and A/H3N2 (-66%); none was statistically significant.

**Conclusions:**

Type- and subtype-specific assessment of influenza VE is needed to identify variations that cannot be differentiated from a measure of VE against all influenza. Type- and subtype-specific influenza VE estimates in Victoria in 2007 and 2008 were generally consistent with strain circulation data.

## Background

Vaccination is the cornerstone of influenza morbidity and mortality prevention and many countries have implemented publicly funded influenza vaccination programs for nationally defined high-risk groups [1]. As part of its National Immunisation Program, in 2007 and 2008 the Australian Government provided free influenza vaccination to all Australians aged 65 years and over, and all Aboriginal and Torres Strait Islander people aged 50 years and over or aged 15-49 years with medical risk factors [2]. Influenza vaccination was also recommended, but not funded, for: individuals aged six months or older with conditions predisposing to severe influenza, people who may potentially transmit influenza to those at high risk of complications from influenza, people providing essential services and travellers. Individual industries are also advised to consider the benefits of offering influenza vaccine in the workplace.

Only split virus and subunit trivalent inactivated influenza vaccines are available for use in Australia against two type A strains (one of each subtype H1N1 and H3N2) and one type B strain which are frequently replaced due to antigenic drift of circulating viruses [2,3]. The World Health Organization (WHO) conducts biannual consultations to recommend which influenza virus strains should be included in the influenza vaccine for the following northern and southern hemisphere seasons [4]. The influenza virus compositions of the 2007 season vaccine were: A/New Caledonia/20/99(H1N1)-like virus; A/Wisconsin/67/2005(H3N2)-like virus; and B/Malaysia/2506/2004-like virus (of the B/Victoria/2/87 lineage) [5] and in 2008 were: A/Solomon Islands/3/2006 (H1N1)-like virus; A/Brisbane/10/2007 (H3N2)-like virus; and B/Florida/4/2006-like virus (of the B/Yamagata/16/88 lineage) [6].

Regular evaluation of vaccination programs by assessment of effectiveness of vaccine to prevent disease is important, particularly for influenza where vaccines often change seasonally. Whilst clinical trials are the ideal method for establishing vaccine efficacy, properly designed observational studies provide a reliable and more practical means of calculating vaccine effectiveness (VE) under field conditions [7,8].

Victoria is Australia's second most populous state with a temperate climate and an annual influenza season that usually occurs between May and September. Here we describe assessment of the effectiveness of seasonal influenza vaccine against laboratory confirmed influenza infection over two consecutive influenza seasons (2007 and 2008) using a test negative case control study design applied to a general practitioner (GP) sentinel surveillance network. We have previously applied this method to assess seasonal influenza VE against any laboratory confirmed influenza [9] but here estimate the type- and subtype-specific protection given by each seasonal influenza vaccine. To our knowledge type and subtype VE estimates have not previously been conducted for a southern hemisphere season.

## Methods

### Sentinel surveillance

A sentinel general practice surveillance program for influenza-like illness (ILI) and laboratory confirmed influenza has been conducted in Victoria by the Victorian Infectious Diseases Reference Laboratory (VIDRL) and the Victorian Government Department of Health since 1998. The program is comprised of a network of GPs throughout Victoria who receive continuing professional development points from the Royal Australian College of General Practitioners and the Australian College of Rural and Remote Medicine for their participation. Each week during the influenza season, GPs report cases of ILI as a proportion of total patients seen. Consistent with established criteria, ILI was defined as history of fever, cough and fatigue/malaise [10]. The GPs were also asked to collect a nose and throat swab from patients presenting with ILI within four days of symptoms onset and forward to VIDRL for testing. Additional data on the patient's age, sex, date of symptom(s) onset, whether vaccinated and date of vaccination were collected on the test request form. In 2007, 50 GPs participated in the sentinel surveillance program which operated for 34 weeks from 12 March (week 11) to 4 November (week 44) inclusive. There were 67 GPs in the 2008 program which operated for 31 weeks from 14 April (week 16) to 16 November (week 46). The program commenced earlier in 2007 to accommodate a pilot varicella-zoster virus infection sentinel surveillance program and finished later in 2008 to enable full capture of ILI patients from a late season commencement.

### Laboratory testing

All nose and throat swab samples were collected using Copan dry swabs placed into virus transport medium. Samples were tested by multiplex polymerase chain reaction (PCR) for influenza A, influenza B, respiratory syncytial virus, picornavirus, parainfluenza virus and adenovirus using a conventional gel based assay [11]. A conserved portion of the matrix gene and haemagglutinin gene were targeted to identify influenza type A and type B viruses respectively, with specific primers for influenza A haemagglutinin H1 and H3 genes used to determine subtype. Specimens were forwarded to the WHO Collaborating Centre for Reference and Research on Influenza for strain typing.

### Ascertainment of cases and controls

Cases and controls were sampled prospectively throughout the study period. A person with ILI who tested positive for influenza was classified as a case whilst a patient with a negative test result, or who was positive for another respiratory virus, was classified as a control. A person selected as a control could become a case for a subsequent separate clinical presentation during the season, but not vice versa. Patients were excluded from the VE analysis if testing did not produce a result.

### Data analysis and calculation of VE

Analyses were conducted using Stata (version 10.0; StataCorp LP). The chi squared test was used to compare proportions, with p < 0.05 considered statistically significant. Patients were excluded from the VE analysis if vaccination status was unknown, if the date of symptom(s) onset was unknown or if there was an interval of greater than four days between symptom onset and specimen collection, based on the decreased likelihood of a positive result after this time [12,13]. For the purposes of analysis, patients were considered not vaccinated if there was less than 14 days between the dates of vaccination and symptom onset.

Vaccine effectiveness was defined as [(1 - odds ratio) × 100%] where the odds ratio is the odds of laboratory confirmed cases being vaccinated divided by the odds of test negative controls being vaccinated. In the test-negative case control design, the odds ratio estimates the incidence density (rate) ratio because controls are selected longitudinally throughout the course of the study (i.e. by 'density sampling') [14,15]. The odds ratio in test-negative case control studies has also been shown to approximate the risk ratio under conditions of varying attack rates and test sensitivity and specificity [16]. Logistic regression was used to calculate odds ratios and 95% confidence intervals (CI) that were adjusted for the confounding variables of age (stratified into the age groups 0-4 years, 5-19 years, 20-49 years, 50-64 years and 65 years and over) and month of specimen collection. Sensitivity analyses were also conducted to determine the effect on VE estimates of: 1) not excluding study participants if more than four days had elapsed between symptom onset and specimen collection; 2) excluding those vaccinated within 14 days of symptoms onset and 3) classifying those vaccinated within 14 days of symptoms onset as vaccinated.

### Ethical considerations

Data in this study were collected, used and reported under the legislative authorisation of the Victorian Health (Infectious Diseases) Regulations 2001 and thus did not require Human Research Ethics Committee approval.

## Results

General practitioners in the sentinel surveillance network saw a total of 182,984 patients during the study period in 2007, of which 1,226 (0.7%) had a reported ILI. The ILI rate peaked in weeks 33 and 34 between 2.0% and 2.2% from a nadir of 0.04% in week 18. In 2008 there were 159,030 patients seen and a total of 876 (0.6%) reported to have an ILI. The weekly rate generally climbed steadily from 0.2% at the start of the 2008 study period in week 18 to a peak of 1.3% in week 35.

General practitioners collected nose and throat swabs for testing from 480 (39%) and 407 (46%) patients with ILI in 2007 and 2008 respectively. Of these, 223 (46%) in 2007 and 117 (29%) in 2008 were positive for influenza. The 2007 season was characterised by a high proportion (58%) of type A/H3N2 influenza cases for which limited strain typing data indicated a generally even split between A/Brisbane/10/2007-like and A/Wisconsin/67/2005-like viruses with a further 17% due to type B and 22% due to A/H1N1 for which all of those typed were the A/Solomon Islands/3/2006-like strain (table 1). A majority (56%) of influenza cases in 2008 were type B with a further 36% due to type A/H3N2 although like 2007, a high proportion of specimens were unable to be recovered or typed (table 1).

**Table 1:** Influenza positive swabs by subtype, year and strain, 2007-2008

Influenza subtype and strain	2007 (%)	2008 (%)
A/H1N1		
A/Solomon Islands/3/2006-like^b^	21 (43)	0
A/New Caledonia/20/99-like^a^	0	0
Not recovered/no result	28^e ^(57)	4 (100)
Total	49 (100)	4 (100)
A/H3N2		
A/Brisbane/10/2007-like^b^	12 (9)	4 (10)
A/Wisconsin/67/2005-like^a^	10 (8)	0
Not recovered/no result	108^e ^(83)	38 (90)
Total	130 (100)	42 (100)
A/subtype not specified	8	6
B		
B/Florida/4/2006-like^b^	2 (5)	1 (2)
B/Malaysia/2506/2004-like^a^	3^c ^(8)	1 (2)
B/Shanghai/361/2002-like	2^d ^(5)	0
Not recovered/no result	30 (81)	63 (97)
Total	37 (100)	65 (100)

^a ^2007 vaccine strain

^b ^2008 vaccine strain

^c ^includes 1 low reactor isolate

^d ^includes 2 low reactor isolates

^e ^1 case positive for A/H1N1 and A/H3N2

Following exclusion of cases for whom vaccination status was unknown, symptom onset or specimen collection dates were unknown or more than four days had elapsed between symptom onset and specimen collection, there were 386 (80%) and 330 (81%) study participants in 2007 and 2008 respectively (table 2). In 2008, a higher proportion of influenza negative patients (17%) compared to influenza positive patients (6%) were excluded because more than four days had elapsed between symptom onset and specimen collection (p = 0.004) whereas in 2007 there was no significant difference (14% and 8%; p = 0.10). There was no statistically significant difference in whether or not study participants had a specimen collected within four days of symptoms onset by age group in either 2007 (p = 0.90) or 2008 (p = 0.09).

**Table 2:** Study inclusion and exclusion criteria by year, 2007-2008

Criteria	2007		2008	
	
	Excluded	Included	Excluded	Included
Inclusion				
Respiratory swabs of ILI patients submitted by GPs	0	480	0	407
Exclusion				
Influenza result unknown	0	480	0	407
Vaccination status unknown	8	472	4	403
Symptom onset to specimen collection interval unknown	41^a^	434	22^a^	384
> 4 days between symptom onset and specimen collection	50^b^	386	54	330

^a ^includes 3 patients with unknown vaccination status

^b ^includes 2 patients with unknown vaccination status

**Figure 1:**
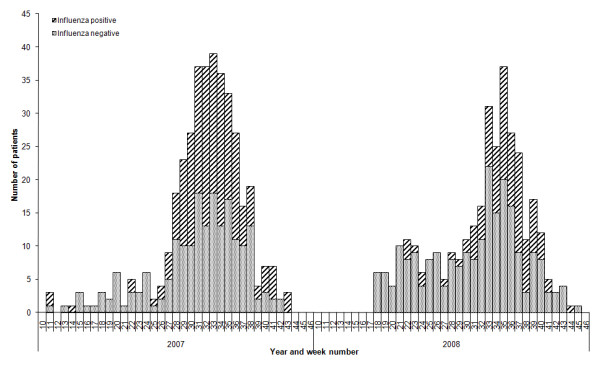
**Case and control recruitment from influenza-like illness (ILI) presentations at sentinel general practices by week and year, 2007-2008**.

An epidemiological curve of influenza negative and influenza positive patients eligible for inclusion in the study (designated as controls and cases respectively) shows an earlier detection of influenza in 2007 compared to 2008, although there was only two weeks' difference in the time from which influenza positive patients were reported for more than three consecutive weeks indicating the start of each season (Figure 1). In addition to a higher number of study participants, the 2007 influenza season was longer (as defined by the number of consecutive weeks in which influenza positive cases were reported) and consisted of a higher proportion of influenza positive study participants (n = 194; 50%) compared to 2008 (n = 106; 32%). The dominant circulating influenza type and subtype varied over the two seasons: 23% of cases in 2007 were A/H1N1, 60% were A/H3N2 and the remainder were type B; the respective proportions in 2008 were 4%, 36% and 57% (table 3).

**Table 3:** Cases and controls by age group, sex, month of swab collection, year and type/subtype, 2007-2008

	2007					2008				
		
	Controls	Influenza cases (%)				Controls	Influenza cases (%)			
				
	(%)	All	A/H1	A/H3	B	(%)	All	A/H1	A/H3	B
Age group (years)										
0-4	7 (4)	7 (4)	3 (7)	4 (3)	0	4 (2)	5 (5)	0	1 (3)	3 (5)
5-19	22 (11)	42 (22)	12 (27)	24 (21)	6 (23)	37 (17)	28 (27)	0	6 (16)	22 (37)
20-49	126 (66)	113 (58)	25 (56)	68 (58)	17 (65)	140 (63)	57 (54)	3 (75)	18 (47)	33 (56)
50-64	28 (15)	22 (11)	4 (9)	14 (12)	2 (8)	30 (13)	11 (10)	1 (25)	9 (24)	1 (2)
≥ 65	9 (5)	10 (5)	1 (2)	7 (6)	1 (4)	13 (6)	4 (4)	0	4 (11)	0
Sex										
Female	89 (46)	96 (49)	20 (44)	62 (53)	12 (46)	105 (47)	55 (52)	1 (25)	19 (50)	34 (57)
Male	103 (54)	98 (51)	25 (56)	55 (47)	14 (54)	119 (53)	51 (48)	3 (75)	19 (50)	26 (43)
Month of swab collection										
March	2 (1)	2 (1)	0	1 (< 1)	1 (4)	0	0	0	0	0
April	5 (3)	1 (< 1)	0	1 (< 1)	0	6 (3)	0	0	0	0
May	14 (7)	1 (< 1)	1 (2)	0	0	28 (13)	3 (3)	0	0	3 (5)
June	13 (7)	4 (2)	1 (2)	3 (3)	0	33 (15)	3 (3)	0	0	3 (5)
July	48 (25)	50 (26)	10 (22)	31 (27)	7 (27)	32 (14)	9 (8)	0	3 (8)	5 (8)
August	66 (34)	94 (48)	30 (67)	56 (48)	4 (15)	69 (31)	42 (40)	4 (100)	17 (45)	21 (35)
September	37 (19)	30 (15)	3 (7)	23 (20)	4 (15)	42 (19)	43 (41)	0	14 (37)	27 (45)
October	7 (4)	12 (6)	0	2 (2)	10 (38)	13 (6)	6 (6)	0	4 (11)	1 (2)
November	0	0	0	0	0	1 (< 1)	0	0	0	0
	
Total	192	194	45	117	26	224	106^a^	4	38	60^a^

^a ^Age unknown for one case

Age group, sex and month of swab collection distributions for controls and cases (including type and subtype strata) are shown in table 3. There was no statistically significant difference in the sex distribution between controls and cases in either 2007 or 2008. In both years, the numbers and proportions of controls and cases were highest in the 20-49 years age group. Influenza type B cases were significantly younger than controls in 2008 (p < 0.001); there were no other statistically significant differences in age distribution between controls and cases. With the exception of subtype A/H1N1 in 2008 for which there were only four cases, stratification by month of swab collection revealed statistically significant differences between cases and controls (range: p < 0.001 to p = 0.02) because of the higher proportion of type A and type B cases identified in August and October 2007 respectively, and subtype A/H3N2 and type B in August/September 2008.

A similar percentage of total study participants were vaccinated in 2007 (19%) and 2008 (17%), although the difference between vaccinated controls and vaccinated cases was generally higher in 2007 (table 4). In 2008 a higher proportion of cases of subtypes A/H1N1 and A/H3N2 were vaccinated compared to controls. In both years the proportion of cases and controls that were vaccinated generally increased with age group. Among the study participants reported as vaccinated, only one control in each year (0.5% in 2007 and 0.4% in 2008) and no cases were vaccinated within 14 days of symptoms onset, for which there was no statistically significant difference.

**Table 4:** Cases and controls by year, age group, vaccination status and type/subtype, 2007-2008

	Total study participants	Total vaccinated (%)	Controls vaccinated (%)	Influenza cases vaccinated (%)			
				
				All	A/H1	A/H3	B
2007							
0-4	14	0	0	0	0	0	0
5-19	64	4 (6)	1 (5)	3 (7)	2 (17)	1 (4)	0
20-49	239	35 (15)	27 (21)	8 (7)	2 (8)	6 (9)	0
50-64	50	20 (40)	13 (46)	7 (32)	2 (50)	2 (14)	1 (50)
≥ 65	19	15 (79)	8 (89)	7 (70)	1 (100)	4 (57)	1 (100)
	
Total	386	74 (19)	49 (26)	25 (13)	7 (16)	13 (11)	2 (8)
	
2008							
0-4	9	1 (11)	0	1 (20)	0	1 (100)	0
5-19	65	4 (6)	2 (5)	2 (7)	0	0	2 (9)
20-49	197	23 (12)	17 (12)	6 (11)	1 (33)	4 (22)	1 (3)
50-64	41	14 (34)	12 (40)	2 (18)	0	1 (11)	1 (100)
≥ 65	17	14 (82)	10 (77)	4 (100)	0	4 (100)	0
	
Total	330^a^	56 (17)	41 (18)	15 (14)	1 (25)	10 (26)	4 (7)

^a ^Age unknown for one case

Following adjustment for month of swab collection and age, there was a statistically significant protective effect of influenza vaccine against all influenza in 2007 (VE = 59%; 95% CI, 25 to 78%) (table 5). The absence of vaccinated cases and controls (table 4) meant VE was unable to be estimated for several of the five age groups so age was collapsed into three variables: children (0-19 years); working age adults (20-64 years); and the elderly (65 years or older). When stratified by age group, the statistically significant association in 2007 was restricted to the 20-64 years age group. Furthermore, when examined by influenza type and subtype, and after adjusting for age group and month of swab collection, the vaccine was found to only be protective at a significant level against the influenza A/H3N2 subtype (VE = 68%; 95% CI, 32 to 85%), for which a statistically significant protective effect was maintained among the working age adults age group only. In 2008, only the unadjusted measure of VE against type B influenza was statistically significant. Receiving vaccine was positively associated with influenza illness for both A/H1N1 and A/H3N2 subtypes after adjustment for age and month of swab collection in 2008 but neither of these associations was statistically significant.

**Table 5:** Crude and adjusted vaccine effectiveness of seasonal vaccine against influenza by year, age group and type/subtype, 2007-2008

	Influenza vaccine effectiveness (95% CI)			
	
	All	A/H1	A/H3	B
2007				
Crude	57 (27, 75)	46 (-28, 77)	64 (29, 81)	76 (-7, 94)
Adjusted^a^				
0-19	-98 (-1906, 80)	-333 (-5401, 66)	-7 (-1850, 94)	Not defined
20-64	64 (29, 82)	48 (-65, 84)	69 (29, 87)	85 (-19, 98)
≥ 65	74 (-283, 98)	Not defined	84 (-156, 99)	Not defined
All ages	59 (25, 78)	27 (-92, 72)	68 (32, 85)	84 (-2, 98)
2008				
Crude	26 (-40, 61)	-49 (-1367, 85)	-59 (-254, 28)	68 (7, 89)
Adjusted^a^				
0-19	-441 (-7774, 63)	Not defined	Not defined	-314 (-6713, 75)
20-64	35 (-56, 73)	-88 (-1936, 83)	-17 (-255, 62)	71 (-32, 93)
≥ 65	Not defined	Not defined	Not defined	Not defined
All ages	9 (-96, 58)	-88 (-1936, 83)	-66 (-349, 39)	49 (-58, 84)

^a ^adjusted for month of swab collection

Sensitivity analyses were conducted to determine the possible effect of assumptions about timing of swab collection and vaccination status on the VE estimates. The effect of not excluding study participants if more than four days had elapsed between symptom onset and specimen collection was a reduction of the adjusted VE point estimates between 7% and 15% in 2007 and between 5% and 35% in 2008. Study participants who were known to be vaccinated within 14 days of symptom onset (one control each in 2007 and 2008) were classified as not vaccinated in the primary analysis. The effect of excluding these cases or classifying them as vaccinated resulted in variations of 0% to 7% around the VE point estimates, but no changes in their relative statistical significance. However, collection of the 'date of vaccination' field only commenced in 2008, in which it was completed for 86 (91%) of the 94 vaccinated study participants. In 2007, only 16 (22%) of the 73 study participants reported as vaccinated had a recorded date of vaccination.

## Discussion

Although there was a low proportion of influenza cases in this study for which strain typing results were available, the statistically significant estimate of 59% effectiveness of influenza vaccine against all influenza in 2007 was generally consistent with Victorian state-wide strain typing data which indicated a partial match of circulating strains to those contained within the vaccine. These data showed A/H3N2 to be the predominant circulating subtype in 2007 (accounting for 56% of the characterised isolates) of which 42% were the A/Wisconsin/67/2005-like (vaccine) strain and the other 58% were the A/Brisbane/10/2007-like strain [17]. When stratified by subtype, the 2007 vaccine was 68% effective (95% CI, 32 to 85%) against A/H3N2 infection and although the A/Brisbane/10/2007-like strain appeared to be the most dominant A/H3N2 strain, the relatively high VE estimate is likely to be explained by the antigenic similarity between the A/Brisbane/10/2007-like and A/Wisconsin/67/2005-like strains [18]. However, stratified analysis did not indicate a significant protective effect of the vaccine against type A/H1N1 or type B infection in 2007, a finding which is supported by apparent mismatch of circulating strains to vaccine strains: 96% of the characterised A/H1N1 isolates were the (non-vaccine) A/Solomon Islands/3/2006-like strain whilst the characterised type B isolates were split between B/Florida/4/2006-like (41%), B/Shanghai/361/2002-like (35%) and B/Malaysia/2506/2004-like (24%) [18].

With a non-significant point estimate of 9%, the adjusted effectiveness of influenza vaccine against all influenza in 2008 was considerably lower than in 2007. The 2008 influenza season in both Victoria and across Australia was of lower magnitude than 2007 and characterised by a higher proportion of cases from influenza type B virus [19,20]. This contrasts with a Western Australian study of the 2008 influenza season, which like Victoria was dominated by type B influenza virus with a late peak, that found a much higher and statistically significant VE point estimate of 58% (95% CI, 9 to 81%) against all influenza [21]. Although this study was restricted to children aged 6-59 months, for whom there is a funded vaccination program in Western Australia, the reason for such a large difference is unclear. Both the sentinel general practice surveillance and other state-wide subtyping data indicated an approximately equal predominance of type A/H3N2 and type B viruses in 2008 [20], although few cases from the sentinel surveillance were able to be strain typed. Crude analysis suggested that the vaccine was 68% effective at a statistically significant level against type B infection, although after adjustment was 49% and not significant. This finding is generally consistent with strain typing data for isolates from across Victoria in which 42% were the vaccine B/Florida/4/2006-like strain and 58% were B/Malaysia/2506/2004-like, between which there was little antigenic similarity given their different lineages (B/Yamagata/16/88 and B/Victoria/2/87 respectively) [19,20]. Strain typing of isolates sourced from elsewhere in Victoria indicated that circulating A/H3N2 was exclusively the A/Brisbane/10/2007-like strain and there was very little circulation of any A/H1N1 strains.

This study demonstrates the importance of conducting type- and subtype-specific assessment of influenza VE given the considerable variation that cannot be differentiated from a measure of VE against all influenza, despite what strain typing of circulating isolates may suggest about vaccine match/mismatch. A Canadian study that measured influenza VE at the trivalent component level during the 2006-2007 northern hemisphere season also observed wide variation between type- and subtype-specific adjusted VE point estimates from 12% to 92% [22], whilst two other observational studies in Wisconsin, United States of America (USA) and Canada also found type-specific variation of VE point estimates from -35% to 58% and 58% to 70% respectively [23,24]. However, stratification of cases to assess type- and subtype-specific influenza VE compromises power as evidenced in this and the Canadian and USA studies. Insufficient power also compromised the ability of our study to generate more precise age group-specific estimates of VE, particularly in 2008 despite the collapse of five age groups into three. This was especially evident for those aged ≥ 65 years (the main risk group eligible for vaccination) in which a protective - but not statistically significant - effect against A/H3N2 influenza was demonstrated in 2007 but had too few cases to generate any VE estimates in 2008, highlighting a previously recognised limitation that the system is best suited to estimating VE amongst working age adults who comprise the majority of the surveillance population [9]. Thus, whilst the program functions well as a representative surveillance system in assessing magnitude and duration of influenza seasons, further recruitment of sentinel GPs may be required to sufficiently power VE calculations, particularly during seasons of low magnitude or a dominant subtype.

A further limitation of this study is that the analysis has not controlled for the potential confounding effect of chronic or co-morbid conditions that are indicated for influenza vaccination. Several Canadian observational studies for which the specific confounding effect of co-morbid conditions was reported resulted in variations of the adjusted type- and subtype-specific VE estimates against seasonal influenza about the crude estimate of -23% to 7% [22,24], and an increase of 15% on the crude seasonal VE against pandemic (H1N1) 2009 influenza [25]. Whilst the confounding effect of co-existing chronic medical conditions on VE estimates may be modest and variable, these data will be included in the patient questionnaire and analysis in future seasons as a single variable. Pooling of confounders has been shown as unlikely to result in residual confounding [26].

Although clinical trials are the ideal method to assess vaccine efficacy, ethical, practical and financial considerations have lead to the emergence of observational studies - in particular case control studies such as this one - to routinely assess influenza VE [22,24,27-29]. However, inherent in observational study designs are biases that should be considered when interpreting and generalising the results to other populations. This study used test-negative control subjects which modelling, assuming no bias, has shown generally slightly underestimates the true VE under most conditions of sensitivity, specificity and influenza to non-influenza ILI attack rates [16] but was higher than traditional control subjects when assessed over three consecutive seasons [27]. Another consideration is the sampling frame of attendees of general practices, for which a high proportion are working-age adults probably representing the mid-range of the clinical spectrum of influenza. More severe presentations (particularly among children and the elderly) are more likely to present to hospitals whilst asymptomatic or mild infections, estimated to be 34% [12], will not present to any medical facility. It is difficult to speculate how exclusion of cases from the peripheries of the clinical spectrum might affect the VE estimates, but highlights the importance of interpreting these results in the context of medically attended ILI in the general practice setting.

Ascertainment bias of influenza status within the study has been minimised by laboratory testing of all study participants with an assay that is at least 90% sensitive and 100% specific for influenza [11], and censoring of observations for which there was greater than four days between onset and specimen collection. Other factors, such as consistency of respiratory specimen collection, are difficult to quantify but may influence VE estimates. Furthermore, participants' illness and vaccination status are only known for the current season and don't account for cross-protection or prior immunity provided by previous vaccination or influenza infection.

## Conclusion

We have applied a test negative case control study design to an established sentinel surveillance system to assess type- and subtype-specific effectiveness of influenza vaccine, which as yet is not routinely undertaken elsewhere in Australia. We found that VE differed by year, influenza type and subtype. Our analysis supplements existing epidemiological and immunological data about seasonal influenza and vaccination to assist with evaluation of the influenza vaccination program.

## Abbreviations

(VE): vaccine effectiveness; (GPs): general practitioners; (PCR): polymerase chain reaction; (WHO): World Health Organization; (ILI): influenza-like illness; (VIDRL): Victorian Infectious Diseases Reference Laboratory; (CI): confidence interval; (USA): United States of America

## Competing interests

The authors declare that they have no competing interests.

## Authors' contributions

JF conducted the data analysis and wrote the paper. KG coordinated recruitment, operation and data management of the surveillance system, undertook data analysis and reviewed and approved the final draft. GP undertook the laboratory testing and reviewed and approved the final draft. HK conceived and designed the study and reviewed and approved the final draft. All authors read and approved the final manuscript.

## Pre-publication history

The pre-publication history for this paper can be accessed here:

http://www.biomedcentral.com/1471-2334/11/170/prepub
